# The role of oxidative stress in diabetes mellitus-induced vascular endothelial dysfunction

**DOI:** 10.1186/s12933-023-01965-7

**Published:** 2023-09-02

**Authors:** Ying An, Bu-tuo Xu, Sheng-rong Wan, Xiu-mei Ma, Yang Long, Yong Xu, Zong-zhe Jiang

**Affiliations:** 1 Department of Endocrinology and Metabolism, The Affiliated Hospital of Southwest Medical University, Luzhou, Sichuan 646000 China; 2 Metabolic Vascular Disease Key Laboratory of Sichuan Province, Luzhou, Sichuan 646000 China; 3 Sichuan Clinical Research Center for Nephropathy, Luzhou, Sichuan 646000 China; 4 Faculty of Chinese Medicine, Macau University of Science and Technology, Avenida Wai Long, Taipa, Macau, China

**Keywords:** Reactive oxygen species, Oxidative stress, Endothelial cells, Diabetic vascular disease

## Abstract

Diabetes mellitus is a metabolic disease characterized by long-term hyperglycaemia, which leads to microangiopathy and macroangiopathy and ultimately increases the mortality of diabetic patients. Endothelial dysfunction, which has been recognized as a key factor in the pathogenesis of diabetic microangiopathy and macroangiopathy, is characterized by a reduction in NO bioavailability. Oxidative stress, which is the main pathogenic factor in diabetes, is one of the major triggers of endothelial dysfunction through the reduction in NO. In this review, we summarize the four sources of ROS in the diabetic vasculature and the underlying molecular mechanisms by which the pathogenic factors hyperglycaemia, hyperlipidaemia, adipokines and insulin resistance induce oxidative stress in endothelial cells in the context of diabetes. In addition, we discuss oxidative stress-targeted interventions, including hypoglycaemic drugs, antioxidants and lifestyle interventions, and their effects on diabetes-induced endothelial dysfunction. In summary, our review provides comprehensive insight into the roles of oxidative stress in diabetes-induced endothelial dysfunction.

## Introduction

Diabetes mellitus (DM) is one of the biggest threats to worldwide public health and leads to a 2–3-fold increased risk of all-cause mortality in individuals and reduced world life expectancy [1]. As a metabolic disease, DM is characterized by chronic hyperglycaemia, hyperlipidaemia, insulin resistance, and hyperinsulinaemia, which cause various complications, including macrovascular and microvascular lesions [2]. The vascular endothelium, which is a monolayer of flattened endothelial cells on the surface of blood vessels [3], plays a pivotal role in vascular physiologic function by regulating vascular permeability, cell adhesion, and the migration and proliferation of smooth muscle cells [4]. Vascular endothelial dysfunction, which is characterized by decreased endothelium-dependent vasodilation, chronic inflammation, hyperpermeability, leukocyte adherence and cell ageing, is the initial stage of vasculopathy and a vital prognostic indicator of diabetic vascular complications [5, 6].

Oxidative stress is an unbalanced redox status that is characterized by the overproduction and accumulation of reactive oxygen species (ROS) and disability of antioxidant systems in cells or tissues [7]. In diabetic patients, vascular endothelial cells are commonly damaged by oxidative stress, which destroys endothelial junctions, increases intravascular permeability, and ultimately contributes to the development of macrovascular and microvascular diseases [8].

Literature retrieval was conducted with PRISMA criteria in Pubmed and Embase databases (as of Mar 30, 2023). The search term for PubMed is: (“Diabetes Mellitus” [Mesh] OR Diabetes Mellitus, Type 2 [Title/Abstract] OR Diabetes Mellitus, Type 1 [Title/Abstract] OR Type 2 Diabetes [Title/Abstract] OR Type 1 Diabetes [Title/Abstract]) AND (“Oxidative Stress” [Mesh] OR Stress, Oxidative [Title/Abstract] OR Reactive Oxygen Specifications [Title/Abstract] OR Oxygen Specifications, Reactive [Title/Abstract] OR Antioxidative Stress [Title/Abstract] AND (“Endohelium” [Mesh] OR endothelial [Title/Abstract] OR vacuum function [Title/Abstract] OR endothelial function [Title/Abstract]), Embase also uses similar search terms. The articles related to the role of oxidative stress in vascular endothelial dysfunction induced by diabetes were included. The exclusion criteria were: (1) comments or meeting abstracts; (2) unable to obtain the original text; (3) published between 1997 and 2023; (4) duplicated publications. Finally, 162 references were obtained in this study.

## Sources of ROS production in diabetic vascular endothelial cells

**Fig. 1:**
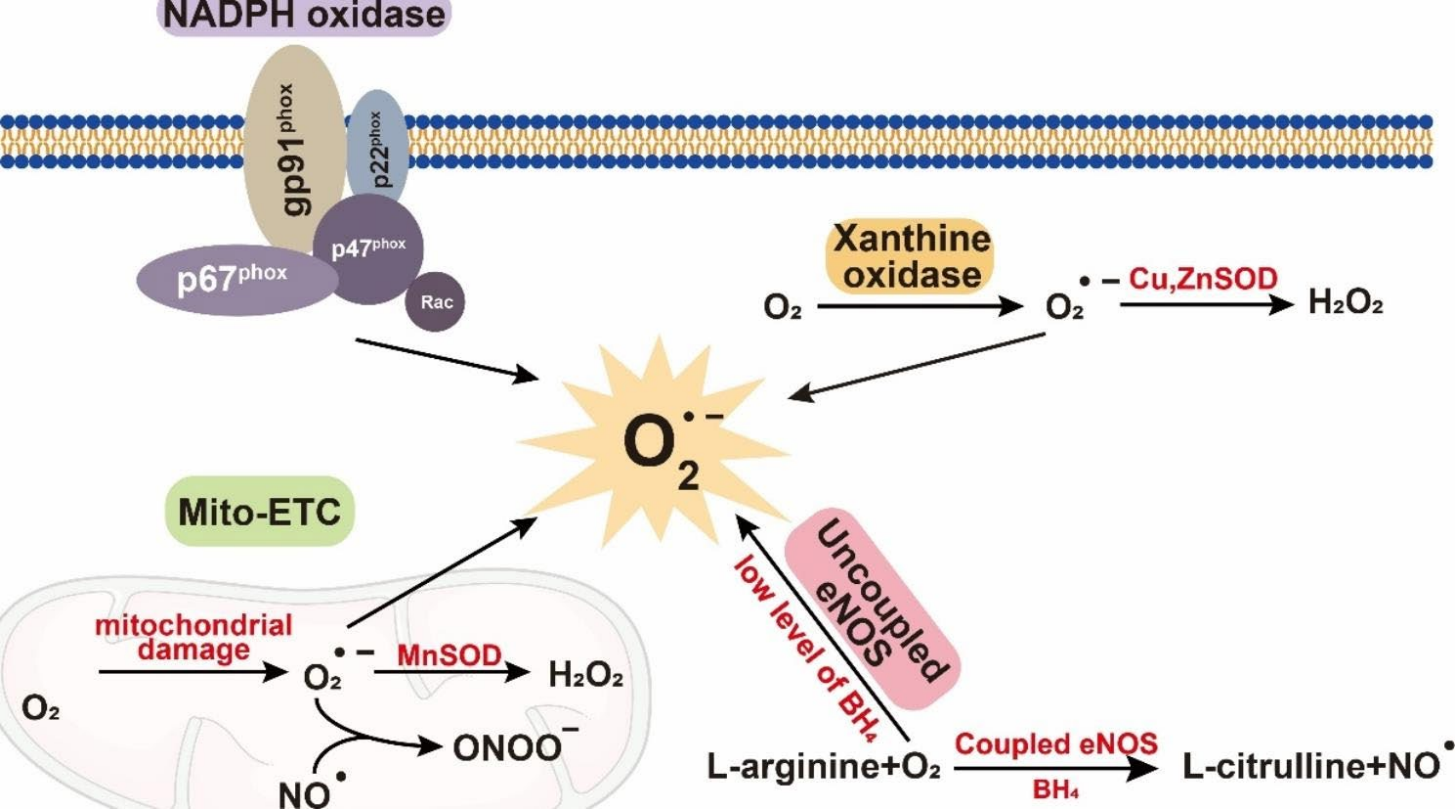
The sources of ROS in diabetic vascular complications. The main sources of ROS in diabetic vascular complications including Mitochondrial electron transport chain, NADPH oxidase, Xanthine Oxidase, and uncoupled endothelial nitric oxide synthase (eNOS), which lead to increased generation of superoxide (O_2_^• −^). Abbreviations: ROS, reactive oxygen species; H_2_O_2_, hydrogen peroxide; ONOO^−^, peroxynitrite; NO^•^, Nitric oxide; Mito-ETC, mitochondrial electron transport chain; BH4, Tetrahydrobiopterin

The major sources of ROS in diabetic blood vessels include the mitochondrial electron transport chain (ETC), nicotinamide dinucleotide phosphate (NADPH) oxidase, xanthine oxidase (XO) and uncoupled endothelial nitric oxide synthase (eNOS) [9](Fig. 1).

### Mitochondrial electron transport chain (Mitochondrial ETC)

 The mammalian mitochondrial ETC consists of complexes I-IV, the electron transporter ubiquinone and cytochrome c. In physiological conditions, some electrons are directly transferred to O_2_ to generate ROS at different sites of ETC complexes [10]. In high glucose-treated bovine aortic endothelial cells, hyperglycaemia induced the overproduction of ROS through an increase in Tricarboxylic acid (TCA) cycle-derived ROS-generating substrates, which could be reversed by incubation with thenoyltrifluoroacetone (TTFA), an inhibitor of complex II, but not rotenone, an inhibitor of complex I [11]. In addition, adenoviral-mediated overexpression of manganese superoxide dismutase (MnSOD), the mitochondrial form of superoxide dismutase, could reduce the proton gradient and inhibit high glucose-induced ROS overproduction in endothelial cells [12]. In a clinical study, it was reported that increased mitochondrial fission in the endothelial cells of diabetic patients contributed to endothelial dysfunction by increasing ROS, suggesting a pivotal role of mitochondrial-derived ROS in diabetes-induced vascular disease [13].

### NADPH oxidase (Nox)

Nox-generated ROS are the leading cause of reduced NO bioavailability in blood vessels. The subunits gp91phox, p22phox, p67phox, p47phox, and Rac1 make up the Nox complex, which generates superoxide radicals by electron transfer to O_2_ [14]. Monocyte Nox is activated by enhanced p22phox expression and p47phox translocation, which is the basis of oxidative stress in T2DM patients [15]. ROS derived from the Nox subtypes Nox1, Nox2, and Nox4 lead to vascular dysfunction in diabetic retinopathy, and Nox5-derived ROS increase the incidence of abdominal aortic aneurysms in diabetic patients [16, 17]. Surprisingly, Nox4 has a protective role in diabetic atherosclerosis [18]. The latest study found that hyperglycaemia and severe acute respiratory syndrome coronavirus 2 (SARS-CoV-2) spike protein induce oxidative stress and apoptosis in endothelial cells by activating the angiotensin-converting enzyme 2 (ACE2)-Nox axis [19].

### Xanthine oxidase (XO)

XO is a crucial enzyme in purine metabolism that oxidizes hypoxanthine to xanthine and then produces uric acid. XO directly transfers electrons to O_2_ to generate O_2_^• −^ and H_2_O_2_ [20]. Renal damage caused by diabetes increases the expression of XO, which produces ROS in endothelial cells, disturbs endothelial homeostasis and causes proteinuria. In contrast, topiroxostat, a nonpurine-specific XO inhibitor, restores impaired glomerular permeability and safeguards glomerular endothelial function. According to this study, diabetic nephropathy is significantly influenced by oxidative stress that is mediated by XO activation [21].

### Uncoupled endothelial nitric oxide synthase (eNOS)

As a subtype of nitric oxide synthase (NOS), eNOS is mainly expressed in endothelial cells and catalyses the conversion of L-arginine, O_2_ and NADPH-derived electrons into NO and L-citrulline. Under physiological conditions, eNOS maintains normal vasodilation and blood pressure and inhibits atherosclerosis through endothelial-derived NO synthesis [22]. However, in diabetic conditions, the uncoupling of eNOS in diabetic vascular endothelial cells leads to the overproduction of superoxide anion (O_2_^• −^), which decreases NO availability, impairs the endothelium and ultimately increases superoxide in blood vessels [23].

## Mechanisms of ROS-induced endothelial dysfunction in diabetic vascular complications

Angiopoietin-1 (Ang-1), endothelial cell-selective adhesion molecule (ESAM), endothelin-1 (ET-1) and other factors regulate endothelial cell function by directly interacting with endothelial cells. Plasma levels of ET-1, which is a vasoconstrictor peptide generated by endothelial cells, are increased in diabetes patients, and ET-1 overexpression directly inhibits eNOS and NO release [24]. Additionally, overexpression of ET-1 promotes the development of type 1 diabetes-associated atherosclerosis, perivascular oxidative stress and inflammation via Nox1 [25]. ESAM belongs to the immunoglobulin superfamily and is produced in vascular endothelial cells. ESAM is crucial for regulating tight endothelial junctions, endothelial permeability, and angiogenesis [26]. Serum ESAM was increased in T2D patients, positively correlated with malondialdehyde (MDA) levels and negatively correlated with catalase activity. ESAM levels were higher in T2D patients with increased oxidative stress [27]. As an endothelium-specific protective factor, Ang-1 contributes to vascular integrity by activating the endothelial cell tyrosine kinase receptor TIE-2. Hyperglycaemia and advanced glycation end products (AGEs) disrupt Ang-1-TIE-2 signalling, inducing the nuclear translocation of FoxO1 and increasing Ang-2 production in endothelial cells. This process leads to vascular instability, inflammatory reactions, and endothelial cell death [28, 29]. Protecting endothelial injury is important for preventing vascular complications in patients with DM. In diabetic conditions, a number of oxidative stress inducers block these endothelial function-related factors by triggering oxidative stress, which results in endothelial dysfunction.

### Hyperglycaemia

**Fig. 2:**
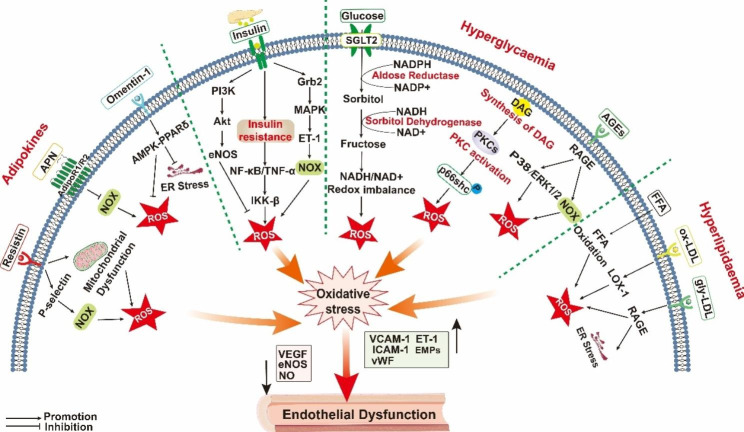
Mechanisms of ROS-induced endothelial dysfunction in diabetic vascular complications. In the diabetic state, oxidative stress induced by hyperglycaemia, hyperlipidaemia, increased resistin and insulin resistance leads to physiological dysfunction of endothelial cells. It accelerates the occurrence and development of diabetic vascular complications through different mechanisms. Abbreviations: ROS, reactive oxygen species; APN,adiponectin; Nox, Nicotinamide adenine dinucleotide phosphate oxidase; ER, endoplasmic reticulum; MAPK, mitogen-activated protein kinase; PPARδ, peroxisome proliferator-activated receptor δ; PI3K, phosphatidylinositol 3 -kinase; Akt, protein kinase B; PKC, protein kinase C; NF-κB, nuclear factor kappa B; TNF-α, tumor necrosis factor α; IKK, inhibition of the NF-κB (IκB) kinase; Grb, Growth Factor Receptor-Bound Protein; SGLT2, Sodium-glucose Transporter2; DAG, diacylglycerol; FFA, free fatty acid; ERK1, Extracellular Signal Regulated Kinase 1; AGEs,advanced glycation end products; RAGE, receptor for AGEs; glyLDL, glycated low-density lipoproteins; ox-LDL, oxidized low-density lipoproteins; LOX-1, Lectin-like ox-LDL receptor-1; ET-1, Endothelin-1; EMPs, Endothelial microparticles; VCAM-1, Vascular cell adhesion molecule-1; VEGF, Vascular endothelial growth factor; VWF, Von Willebrand factor; eNOS, endothelial nitric oxide synthase; ICAM-1, Intercellular cell adhesion molecule-1. Figure was drawn by the authors using Adobe Illustrator tools

The current mechanisms associated with hyperglycaemia-induced diabetic vascular complications mainly focus on the following four pathways: the formation of AGEs, the activation of PKC, the increase in flux through the poly-alcohol pathway, and the increase in flux through the hexosamine pathway. These pathways can lead to oxidative stress-induced endothelial damage in diabetes through the overproduction of ROS [11, 12](Fig. 2).

Nonenzymatic reactions of monosaccharides such as glucose, glyceraldehyde, and fructose with protein amino groups, lipids, and nucleic acid amino groups can result in the formation of senescent macroprotein derivatives, which are called AGEs [30, 31]. Under hyperglycaemic conditions, increased AGEs in plasma induce ROS production by binding to the cell surface receptor for AGEs (RAGE) [32–34]. Endothelial homeostasis becomes unbalanced as a result of AGEs binding to RAGE in endothelial cells, which increases mitogen-activated protein kinase (MAPK) and enhances the production of lysyl oxidase and ET-1 under the control of nuclear factor kappa B (NF-κB) and activator protein-1 (AP-1) [35]. Endothelial function is inversely linked with plasma levels of AGEs in patients with T2DM and coronary atherosclerosis. The activation of p38 and ERK1/2 by AGEs in human coronary artery endothelial cells (HCAECs) increases oxidative stress and decreases eNOS expression, which in turn causes endothelial dysfunction [36]. High expression of Peroxidasin (PXDN) was found in db/db mice with impaired endothelium-dependent relaxation. In human umbilical vein endothelial cells (HUVECs), the upregulation of Nox2 expression by AGE-RAGE increased ROS levels, which promoted PXDN and hypochlorous acid (HOCl) expression. PXDN-derived HOCl promotes AGE-induced endothelial dysfunction in diabetes by blocking Akt-eNOS phosphorylation and NO release [37]. Endothelial microparticles (EMPs), which are biomarkers of endothelial injury, are increased in the serum of patients with T2DM. AGEs/RAGE induce the release of EMPs through the Nox-derived ROS pathway, which is critical for the molecular mechanism of EMP release in diabetic vascular diseases [38].

In hyperglycaemia, diacylglycerol is synthesized de novo from dihydroxyacetone phosphate, which is an intermediate product of glycolysis that then activates PKC [39]. In response to high glucose stimulation, 8-oxoguanine glycosylase (OGG1), an oxidative DNA repair enzyme, is reduced, which induces increased ROS production by endothelial cells via the PKC/Nox pathway [40]. Different PKC isoforms (PKC-α, PKC-β and PKC-δ) are involved in vascular endothelial functional impairment through their different mechanisms in the diabetic state [41]. First, selenoprotein S (SELENOS) can protect human aortic endothelial cells (HAECs) against multifactor-induced oxidative stress by modulating the diabetic vascular endothelial microenvironment, resulting in PKC-α inhibition and indirect activation of the PI3K/Akt/eNOS signalling pathway. SELENOS may become a new target for preventing and treating macrovascular complications in diabetes [42]. Second, PKC-β activation promotes endothelial cell dysfunction caused by dysregulation of the IL-18/IL-18-binding protein pathway in diabetic patients, leading to increased vascular cell adhesion molecule-1 (VCAM-1) expression, monocyte adhesion and accelerated atherosclerotic plaque formation [43]. In addition, high glucose activates ROS by activating the PKCβ-p66shc signalling pathway, leading to NF-κB activation, increased intercellular cell adhesion molecule-1 (ICAM-1) expression and reduced eNOS expression, which induces oxidative stress damage in endothelial cells and diabetic foot tissue [44]. Third, hyperglycaemia-induced PKC-δ-specific activation in endothelial cells reduces the effect of vascular endothelial growth factor (VEGF), and ablation of PKC-δ in endothelial cells improves blood flow reperfusion and lateral limb angiogenesis in diabetic ischaemic limbs [45].

Under hyperglycaemic conditions, hexokinase is saturated, resulting in more glucose entering the polyol pathway, which consists of only two steps, the first of which is the transformation of glucose to sorbitol by the activity of aldose reductase (AR). This process depletes NADPH, reducing intracellular peroxide clearance and increasing the levels of oxidative stress. The second step depletes NAD+ to convert sorbitol to fructose in the presence of sorbitol dehydrogenase. This pathway is thought to be the main pathway leading to the NADH/NAD + redox imbalance in diabetes [46–48]. During hyperglycaemia, ROS produced by AR can accelerate thrombus formation in diabetic vessels by activating c-Myc, inhibiting miRNA-24 and enhancing von Willebrand factor (VWF) release from endothelial cells in diabetic patients [49]. On the other hand, AR inhibitors inhibit high glucose-induced ROS production and apoptosis in HUVECs, restore Sirtuin 1 (SIRT1) expression and phosphorylation of AMPKα1, and reduce mTOR phosphorylation, preventing hyperglycaemia-induced endothelial cell death and dysfunction [50]. In addition, the DNA binding and transcriptional activity of RUNX2 plays a vital role in maintaining endothelial cell function and promoting angiogenesis. In contrast, endothelial cells exposed to high glucose have inhibited RUNX2 activity via the AR pathway, inhibiting the latter’s DNA-binding activity and causing diabetic vascular dysfunction [51].

In addition, hyperglycaemia leads to oxidative stress by increasing mitochondrial superoxide production, thereby increasing hexosamine pathway (HBP) activity by 2.4-fold [52]. HBP is another glycolysis pathway that accounts for a tiny proportion of glucose metabolism. The intermediate product of glycolysis is fructose 6-phosphate, which is formed by the action of fructose-6-phosphate amidotransferase (GFAT) on glucosamine 6-phosphate; through acetylation via UTP, UDP-N acetylglucosamine (UDP-GlcNAc) is formed, which generates O-linked N-acetylglucosamine (O-GlcNAc) in the presence of O-GlcNAc transferase [53]. In the diabetic state, an increase in O-GlcNAc impairs endothelial cell migration and tube formation by reducing the activity and phosphorylation of Akt at serine 473 [54]. During hyperglycaemia, elevated O-GlcNAc modification of the transcription factor specificity protein 1 (SP1) increases ICAM-1 expression in HUVECs and rat retinal capillary endothelial cells and upregulates VEGF-A expression in retinal cells [55, 56]. The precise role of the HBP in the vascular problems of diabetes requires more research.

### Hyperlipidaemia

High blood levels of free fatty acids (FFAs), high triglyceride levels and abnormal lipoproteins are the main manifestations of dyslipidaemia in patients with T2DM [57]. Increased oxidation of FFAs in aortic endothelial cells leads to increased peroxide production by the mitochondrial ETC, which activates ROS and inactivates two important antiatherosclerotic enzymes: prostacyclin synthase and eNOS [58]. Patients with diabetes have elevated glycated low-density lipoproteins (glyLDL) and oxidized low-density lipoproteins (ox-LDL). An increase in glyLDL or ox-LDL significantly reduces oxygen consumption and mitochondrial ETC enzyme activity of mitochondrial ETC complexes I, II/III and IV in porcine aortic endothelial cells, leading to oxidative stress in vascular endothelial cells and the development of diabetic vascular complications [59]. Lectin-like ox-LDL receptor-1 (LOX-1) is the primary receptor of ox-LDL in endothelial cells. High glucose induces oxidative stress through the activation of Nox2 and p47phox, which causes the upregulation of LOX-1 and downregulation of eNOS in HAECs [60]. LOX-1 activation further induces ROS production, promotes ox-LDL uptake, reduces NO release from endothelial cells, induces the expression of ET-1, AT1 receptors and cell adhesion molecules, and enhances vascular homeostasis disorders and endothelial dysfunction [61]. In human endothelial cells (HECs), GlyLDL treatment upregulates the level of RAGE, which increases endoplasmic reticulum stress (ERS) and oxidative stress. The increased levels of ERS and oxidative stress lead to the activation of the NF-κB and p38 MAPK signalling pathways, which increase monocyte adhesion to endothelial cells by upregulating the expression of VCAM-1 [62].

### Adipokines

Adipokines, including adiponectin (APN), omentin and resistin, act directly on endothelial cells through blood circulation.

APN binds to the membrane receptors AdipoR1, AdipoR2, and T-cadherin and is mainly released by adipose tissue. It has physiological effects such as hypoglycaemia, hypolipidaemia, anti-inflammation, and insulin sensitivity [63, 64]. In the retinal vascular system, APN induces the vasodilation of small retinal arteries by binding to AdipoR1/AdipoR2 in retinal endothelial cells (RECs) in an AMPK-dependent manner through activation of eNOS to release NO [65]. In a hyperglycaemic state, reduced expression of T-cadherin receptors in the retinal vascular endothelium leads to APN deficiency, causing increased vascular permeability and a significant increase in VCAM-1, which damages endothelial cells [66]. Exogenous supplementation with APN acts as a potent antioxidant to suppress oxidative stress by inhibiting gp91phox and nitrotyrosine protein, which reduces the production of aortic inflammatory molecules (tumour necrosis factor α[TNF-α], IL-6 and ICAM-1) and improves endothelial dysfunction in obese/diabetic patients [67]. Hypoadiponectinemia also promotes oxidative/nitrosative stress by promoting the expression of gp91phox and inducible NOS, which induces NOD-like receptor family pyrin domain-containing 3 (NLRP3) inflammatory vesicle activation and endothelial dysfunction in diabetic vascular tissue [68].

Omentin, which is also known as intelectin, is expressed not only in vascular cells but also mesothelial cells, airway goblet cells and visceral (omental and epicardial) fat, small intestine, colon, ovaries, and plasma [69]. As a potential target for the treatment of T2DM and atherosclerosis, omentin-1 can ameliorate vascular dysfunction by regulating endothelial cell function, including proliferation, migration and apoptosis [70, 71]. Serum omentin-1 levels are reduced and inversely linked with disease severity in T2DM patients with peripheral vascular disease [72]. Omentin-1 decreases oxidative stress in T2DM animal models by reducing plasma MDA, urinary 8-hydroxy-2′-deoxyguanosine (8-OHdG), vascular superoxide anion and nitrotyrosine levels, further increasing p-eNOS levels and NO bioavailability to protect endothelial cells. Additionally, it lowers periaortic C-reactive protein (CRP) levels, reduces inflammation in perivascular adipose tissue (PVAT), and enhances the anticontractile function of PVAT [73]. Omentin-1 can also directly activate the AMPK-peroxisome proliferator-activated receptor δ (PPARδ) pathway, increasing the phosphorylation of Akt and eNOS and preventing oxidative stress and ERS to protect endothelial cell dysfunction caused by high glucose levels [74].

Resistin is another adipokine that is produced during the catabolic process of adipocytes. In HCAECs, resistin induces mitochondrial dysfunction and cellular oxidoreductase imbalance, leading to excessive ROS production and eNOS reductions. SB-239,063, a specific p38 inhibitor, prevents resistin-induced ROS generation and eNOS downregulation [75]. Resistin upregulates P-selectin and fractalkine in HECs in response to high glucose stimulation, which leads to oxidative stress through Nox-mediated ROS production and increases monocyte adhesion through the activation of NF-κB and AP-1 [76]. T2DM patients have increased serum resistin levels, which directly promote oxidative stress and insulin resistance by increasing serum Dickkopf-1 and urinary 8-iso-PGF2α levels [77].

### Insulin resistance

In the cardiovascular system, insulin can increase the concentrations of NO, ET-1 and ROS by activating downstream signalling pathways. In the context of insulin resistance, elevated insulin concentrations in blood vessels may lead to vascular abnormalities by increasing the secretion of endothelial mediators, such as NO, ET-1 and ROS [78]. Insulin’s antiatherogenic effects are mediated by the PI3K/Akt signalling pathway, resulting in eNOS activation, an increase in NO release, and a decrease in ET-1 production in endothelial cells, all of which result in vasodilation. Reduced expression of haem oxygenase-1 (HO-1), VEGF, and VCAM-1 can enhance blood vessels through antioxidative stress and anti-inflammatory effects. In contrast, insulin activation of the Grb/Shc/MAPK pathway promotes the expression of ET-1 and plasminogen activator inhibitor-1 (PAI-1) and the migration and proliferation of constricted cells, both of which have a proatherogenic effect [79]. Endothelial IR is present in diabetic patients with endothelial dysfunction, which disrupts endothelial insulin signalling through the activation of PKC-β and NF-κB activity [80]. Insulin resistance leads to excessive endothelial superoxide production, severe damage to the aortic wall and accelerated atherosclerosis, and Nox2 is the main source of superoxide [81]. In addition, TNF-α and NF-κB induce insulin resistance and amplify oxidative stress through the IKK-β pathway, leading to endothelial dysfunction in T2DM coronary arteries [82].

### MicroRNAs

**Fig. 3:**
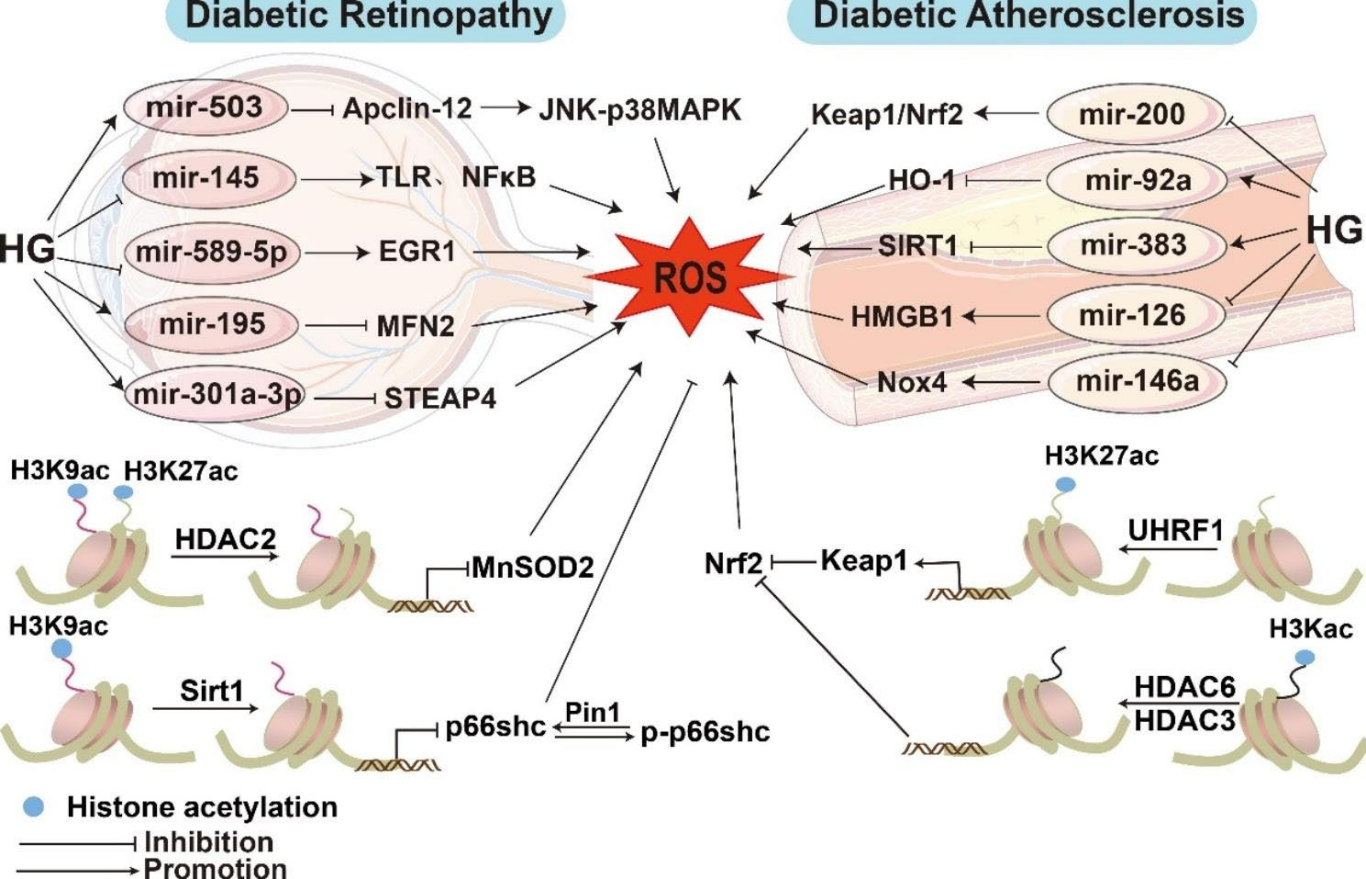
The role of microRNAs and histone acetylation in ROS-induced endothelial dysfunction in diabetic vascular complications. In the diabetic state, oxidative stress induced by epigenetic alterations such as microRNAs and histone acetylation alterations leads to endothelial physiological dysfunction. Abbreviations: ROS, reactive oxygen species; JNK, Jun NH2-terminal kinase; SIRT1, Sirtuin1; Keap, kelch-like ECH-associated protein; Nrf2, Nuclear factor erythroid 2-related factor 2; Pin1, peptidyl-prolyl cis/trans isomerase 1; EGR1, Early Growth Response Protein 1; HO-1, heme oxygenase‐1; HMGB1, high-mobility group box 1; MFN2, mitofusin 2; TLR4, toll-like receptor 4; NF-κB, nuclear factor-κB; HMGB1, high-mobility group box 1;HDAC, histone deacetylase; MnSOD, manganese superoxide dismutase; H3K9, histone 3 lysine 9; UHRF1, ubiquitin-like containing PHD and RING finger domains 1

Noncoding RNAs play a significant role in the pathophysiology of oxidative stress. Noncoding RNAs can be broadly divided into two types: short RNAs, such as microRNAs, and long-stranded RNAs, such as lncRNAs. Here, we will quickly discuss the roles of various microRNAs in the oxidative stress-induced development of diabetic vascular problems [83] (Fig. 3).

Diabetic retinopathy is a typical diabetic microvascular complication, and microRNAs regulate key signalling pathways. The upregulation of miR-195 in the diabetic rat retina decreased mitofusin 2 (MFN2) protein levels, reduced mitochondrial fusion, accelerated oxidative stress-induced damage to RECs in diabetic rats, and increased diabetic retinal vascular permeability [84]. MiR-145 expression is dramatically decreased in high glucose-treated RECs, which leads to high glucose-induced oxidative stress and inflammation in RECs by targeted enhancement of toll-like receptor 4 (TLR4) expression and nuclear translocation of NF-κB p65 [85]. On the contrary, miR-301a-3p is increased in retinal tissue of diabetic retinopathy mouse model and promotes ROS production by inhibiting six-transmembrane epithelial antigen of prostate 4 (STEAP4) [86]. MiR-503 inhibits Apelin-12 expression in high glucose-treated human microvascular endothelial cells and increases inflammation and oxidative stress through the JNK and p38MAPK signalling pathways [87]. Diabetic retinopathy upregulates circ-UBAP2 expression. Knocking out circ-UBAP2 in the miR-589-5p/EGR1 axis alleviates high glucose-induced oxidative stress and vascular dysfunction in human retinal microvascular endothelial cells (HRMECs) by increasing superoxide dismutase (SOD) and glutathione peroxidase activities and nuclear factor erythroid 2-related factor 2 (Nrf2), HO-1, and SOD-1 expression, which provides a promising therapeutic target for diabetic retinopathy [88]. Besides, miR-93 suppression induced by 25-dihydroxyvitamin D3 inhibits the high glucose-induced overproduction of ROS, MDA and ferroptosis in HRMECs [89].

Atherosclerosis is a classic manifestation of diabetic macroangiopathy. As an anti-inflammatory mediator of the vascular system against atherosclerosis, miR-181b levels are reduced in the arteries of diabetic patients. In contrast, AMPK activation increases the levels of miR-181b in endothelial cells and reduces endothelial dysfunction in diabetic mouse arteries by preventing the formation of ROS and vascular inflammation [90]. MiR-200a is involved in regulating redox imbalance, and the suppression of aortic endothelial miR-200a expression under hyperglycaemic conditions increases the expression of kelch-like ECH-associated protein 1 (Keap1), which is linked to Nrf2 binding and leads to the inactivation of Nrf2 antioxidant signalling, thereby inducing diabetic vascular endothelial dysfunction [91]. MiR-146a expression was decreased in endothelial cells stimulated by high glucose and thrombin, resulting in the increase of Nox4 expression, promoting ROS production and endothelial inflammation, which contributes to the pathogenesis of diabetes-related atherosclerosis and vascular damage [92]. MiR-126 was reduced in the aorta of ApoE ^−/−^ mice with diabetes, which induced ROS production and endothelial inflammation by enhancing the expression of high-mobility group box 1 (HMGB1). Overexpression of miR-126 could reverse the decrease in AKT and eNOS phosphorylation caused by high glucose, exerting a protective effect on anti-inflammatory symptoms [93]. In contrast, miR-351 was elevated in the mouse model of T2DM with Atherosclerosis, which inhibited the expression of integrin subunitβ3 and PI3K/Akt pathway, leading to the decline of antioxidant stress capacity and survival rate of aortic endothelial cells [94]. Moreover, miR-92a and miR-383 levels are elevated in the aortic endothelium of db/db mice. MiR-92a inhibition reduces oxidative stress and improves endothelial function by upregulating the expression of HO-1 [95]. Inhibition of miR-383 reduced ROS by upregulating SIRT1, increasing SOD1/CAT levels, and enhancing endothelial cell activity [96].

Besides, miR-34a was elevated in renal tissue of diabetic kidney disease (DKD) and increased the levels of ROS and inflammation in human renal glomerular endothelial cells by inhibiting the expression of autophagy-related gene 4B (ATG4B), which might contribute to the pathogenesis of DKD [97]. Notably, miR-142-5p delivered by high glucose-induced monocyte extracellular vesicles reduced migration and increased ROS production in HUVECs and inhibition of miR-142-5p alleviated inflammation in T1DM mice aortas [98]. In a word, these studies suggest that inhibition or activation of microRNAs could be a potential adjuvants to diabetic vascular therapy.

### Histone acetylation

In recent years, histone acetylation and deacetylation have been shown to impact the epigenetic regulation of cellular transcription and have received increasing attention for their role in diabetic vascular complications. Histone acetyltransferases and histone deacetylases catalyse acetylation and deacetylation, respectively [99]. Histone acetylation is an enzyme-catalysed posttranslational modification that results in a functional alteration in gene transcription in the acetylated histone region. In contrast, histone deacetylases repress gene transcription [100].

An increase in the expression and activation of histone deacetylase 2 (HDAC2) inhibits MnSOD expression by reducing the acetylation levels at histone 3 lysine 9 (H3K9) and H3K27, thereby inducing oxidative stress and apoptosis in ECs under diabetic conditions [101]. The histone deacetylase HDAC3 was also activated in the thoracic aortic endothelium of db/db mice, and its inhibition prevented high glucose- and palmitic acid-induced dysfunction in HUVECs by decreasing Keap1 synthesis to increase Nrf2 levels and downregulate Nox4, which suggests that HDAC3 is a target for the treatment of endothelial dysfunction in T2DM [102]. The HDAC6-specific inhibitor tubastatin A (TS) prevented a high glucose-induced increase in the oxidative/nitrosative stress markers nitrotyrosine and 4-HNE by upregulating the NAD-dependent deacetylase SIRT1 in human retinal endothelial cells (HRECs) [103]. Unlike HDACs, the deacetylase SIRT1 has been reported to play an antioxidant role in diabetes [104]. Overexpression of endothelial-specific SIRT1 suppressed the high glucose-induced senescence indicators p53, p21, and PAI-1 [105]. High glucose increased p66Shc expression by decreasing SIRT1 expression in HRECs, which in turn increased H3K9 acetylation at the binding site of the p66Shc promoter and p53 transcription factor. Moreover, increased phosphorylated p66Shc (p-p66Shc) interacts with peptidyl-prolyl cis/trans isomerase 1 (Pin1) to increase mitochondrial ROS and damage the retinal microvasculature in diabetic rats [106]. Ubiquitin-like containing PHD and RING finger domains 1 (UHRF1), despite being a nonhistone deacetylase protein, was significantly reduced in endothelial colony-forming cells (ECFCs) from type 2 diabetic patients, which promoted Keap1 expression by increasing the level of H3K27Ac and subsequently induced ROS overproduction and apoptosis in diabetic ECs [107]. In conclusion, the active exploration of new histone modifications is particularly important to reduce the severity and risk of vascular complications in diabetes.

## Effect of targeting oxidative stress in diabetes-induced endothelial cell dysfunction

### Hypoglycaemic drugs

In the clinic, glucose-lowering agents such as biguanide, sodium-glucose transporter 2 (SGLT2) inhibitor, dipeptidyl peptidase 4 (DPP-4) inhibitors, glucagon-like peptide 1 (GLP-1) and Thiazolidinediones can not only control blood glucose but also improve endothelial function by targeted regulation of oxidative stress, thus preventing/improving vascular complications of diabetes [108] (Table 1).

Early in 2001, metformin, which is a classic hypoglycaemic drug, was reported to improve insulin resistance and endothelial function in patients with type 2 diabetes at a dose of 500 mg orally twice per day for 12 weeks [109]. In a study of a diabetic mouse model, metformin treatment (300 mg/kg/d) slowed the progression of atherosclerosis by inhibiting DRP1-mediated mitochondrial division [110]. In another study with HUVECs. Metformin treatment (0.5 mM) reduced high glucose-induced ROS production by upregulating OGG1 expression via the AMPKα-Lin-28 pathway [111]. In addition, metformin treatment (100 µmol) ameliorated high glucose and high fat-induced cardiac microvascular endothelial cells (CMECs) injury by upregulating mitochondrial membrane 70 (Tom70) expression to reduce mitochondrial disorders and oxidative stress [112]. However, metformin treatment at a therapeutic concentration for diabetes (1–50 µM) did not affect the oxygen consumption rate of mitochondrial complex I or complex III but still prevented high glucose-induced ROS production in cultured vascular endothelial cells, which indicates that high concentration-mediated mitochondrial protection does not contribute to the antioxidant function of therapeutic concentrations in diabetic patients. In fact, therapeutic doses of metformin (1–50 µM) protect endothelial function by interacting with the orphan nuclear receptor Nur77/NR4A1 to reduce hyperglycaemic-induced ROS [113]. Overall, although a large number of studies have reported the protective effects of metformin on cell or mouse models, the results must be interpreted with considerable caution because the concentrations used in vitro are markedly higher than those in *vivo*.

DPP-4 inhibitors also have protective effects on vascular endothelial cells. For example, saxagliptin attenuates ox-LDL-induced cytokine and vascular adhesion molecule (TNF-α, Il-1β, ICAM-1, VCAM-1) production by inhibiting AP-1 and NF-κB and reduces Nox4 expression, thereby inhibiting the excessive generation of ROS and improving endothelial dysfunction [114]. In contrast, sitagliptin ameliorates high glucose-induced apoptosis by activating AMPKα in HUVECs, effectively preventing high glucose-induced ROS generation and mitochondria-dependent apoptosis [115]. Long-term administration of teneligliptin induced antioxidant responses in HUVECs and increased the expression of downstream target genes of Nrf2, thereby improving oxidative stress. This treatment also reduces high glucose-induced ERS, promotes cell recovery by reducing apoptosis and enhancing cell proliferation, and improves the metabolic memory effect induced by long-term exposure of endothelial cells to high glucose [116]. Concomitant administration of teneligliptin and GLP-1 was more effective than teneligliptin alone in countering ROS production and had more beneficial effects on oxidative stress, endoplasmic reticulum homeostasis, cell proliferation and apoptosis [117].

GLP-1 is a protective target against cardiovascular complications in T2DM. Exenatide protects HUVECs and the microvasculature of diabetic rats from oxidative stress in vitro and in vivo by inhibiting Nox4 and p47phox translocation [118]. In addition, exenatide reduces apoptosis in human retinal vascular endothelial cells (HRVECs) by reducing inflammatory cytokines (IL-1β, TNF-α) and ROS overproduction by lowering sphingosine-1-phosphate receptor 2 (S1PR2) levels [119]. Recombinant human glucagon-like peptide-1 (rhGLP-1) reversed diabetic nephropathy by reducing oxidative stress in glomerular microvascular endothelial cells and protecting the glomerulus and tubules through inhibition of PKC and activation of PKA [120]. Liraglutide ameliorated PA-induced murine islet microvascular endothelial cells with oxidative stress, apoptosis and ET-1 secretion dysfunction [121]. Dulaglutide inhibits the expression of PAI-1 by restoring the expression of SIRT1 and eNOS, restores telomerase activity and finally alleviates high glucose-induced oxidative stress in HRECs [122]. Dulaglutide also increased the expression of SIRT1 to inhibit high glucose-induced NLRP3 inflammasome activation, and down-regulated the expression of NOX4 and ROS generation in HUVECs [123]. Lixisenatide, as a GLP-1 receptor agonist, shows anti-inflammatory and antioxidant effects on FFA-induced endothelial dysfucntion. It inhibits the ROS production and promotes the eNOS phosphorylation and NO production in a dose-dependent manner in FFA-treated HUVECs [124].

Dapagliflozin, canagliflozin and empagliflozin are SGLT2 inhibitors. Dapagliflozin reduces the phosphorylation of ERK1/2 and the activity of cPLA2 in HRMECs induced by high glucose, reducing the production of arachidonic acid and ROS and attenuating apoptosis in HRMECs induced by DM [125]. Additionally, Dapagliflozin also improves endothelial dysfunction by increasing eNOS activity and inhibiting ROS generation via SIRT1 activation in hydrogen peroxide-induced endothelial oxidative stress [126]. In diabetic ApoE^-/-^ mice, canagliflozin was reported to protect endothelial function and reduce the incidence of atherosclerosis by decreasing aortic Nox2 and p22phox expression and urinary 8-OHdG excretion to minimize oxidative stress and considerably reduced aortic ICAM-1 and VCAM-1 expression [127]. Similar to metformin, empagliflozin activates the AMPK signalling pathway, inhibits Drp1S616 phosphorylation, increases Drp1S637 phosphorylation, inhibits mitochondrial fission and subsequently prevents CMECs senescence by inhibiting mitochondrial ROS production, thereby increasing the viability and barrier performance of CMECs. However, empagliflozin causes CMECs to migrate and promotes angiogenesis [128]. In addition, Empagliflozin reduced mitochondrial Ca^2+^ overload and ROS production in high glucose-treated HECs, attenuated endothelial leakage and improves cell viability in response to H_2_O_2_ treatemnt in human brain microvascular endothelial cells (HBMECs) [129]. Notably, the underlying mechanism of SGLT-2 inhibitor-mediated endothelial protection from high glucose is not due to their ability to lower blood glucose but rather to the blockade of the entry of glucose into the endothelium via the endothelial SGLT-2 transporter [130]. In summary, as new drugs marketed in the last few years, SGLT2 inhibitors show promising therapeutic potential in treating diabetic pathological microangiopathy.

Thiazolidinediones such as Pioglitazone and Rosiglitazone which are commonly used in type 2 diabetic patients, are also reported to exhibit antioxidant effect on diabetic vascular disease [131]. Pioglitazone administration restores the endothelial function by inhibiting oxidative stress and increasing nitric oxide levels in aorta of STZ-diabetic rat [132]. As an agonist of peroxisome proliferator-activated receptor-γ (PPARγ), Rosiglitazone was reported to suppress vascular endothelial cell activation and injury by inhibiting mitochondrial oxidative stress and dysfunction through heat shock protein 22 (HSP22)-dependent regulation of PPARγ in diabetes angiopathy [133]. Notably, Rosiglitazone could also protect endothelial cells against glucose-induced oxidative stress via an AMPK-dependent and PPARγ-independent mechanism, in which Rosiglitazone activates AMPK and the latter subsequently prevents hyperactivity of NOX induced by high glucose [134].

**Table 1:** The in vivo and in vitro studies of Hypoglycemic Drugs

Study Type	Hypoglycemic Drugs	Model	Dose	Result	Refs
In vitro	metformin	HUVECs	Metformin(0.5mM, 24 h)	p-AMPK↑ OGG1↑ ROS↓	[111]
	metformin	CMECs	Metformin (10µM, 100µM, 1000µM, respectively for 24 h)	Tom70↑ NO↑ ROS↓ GSH↑	[112]
	DPP-4 inhibitor	Primary HUVECs	Saxagliptin (0.5µM, 1µM, respectively for 12 h) ox-LDL(100 µg/ml for 24 h.)	LOX-1↓ ICAM-1,↓ VCAM-1↓ NOX-4↓	[114]
	DPP-4 inhibitor	HUVECs	teneligliptin (0.1µM, 1.0µM, 3µM respectively for 21days)	NOX-4↓ BAX↓ BCL2↑ ROS↓	[116]
	DPP-4 inhibitor	HUVECs	Sitagliptin(1µM, 48 h)	p-AMPK↑ ROS↓	[115]
	GLP-1	HUVECs	Exenatide(10 µg/ml, 24 h)	S1PR2↓ IL-1β↓ TNF-α↓ ROS↓	[119]
	GLP-1	IMECs	Liraglutide(3nM,10nM,30nM, 100nM respectively for 24 h)	PKA↑ eNOS↑	[121]
	GLP-1	HRECs	Dulaglutide(25nM, 50nM respectively for 24 h)	SIRT1↑ eNOS↑ PAI-1↓	[122]
	GLP-1	HUVECs	Dulaglutide(50nM, 100nM respectively for 48 h)	SIRT1↑ NLRP3↓ NOX4↓	[123]
	GLP-1	HUVECs	Lixisenatide(10nM, 20nM respectively for 24 h)	ICAM-1↓ VCAM-1↓ eNOS↑ ROS↓	[124]
	SGLT2 Inhibitor	HBMECs	empagliflozin (1 µM, for 48 h)	mtROS↓	[129]
	Thiazolidinediones	HUVECs	Rosiglitazone(1 to 20 µM respectively for 48 h)	AMPK↑ NOX↓ ROS↓	[134]
In vivo	metformin	STZ-induced male ApoE^−/−^mice,	Metformin (300 mg/kg/day) by drinking water	p-AMPK↑ Drp1↓ mitochondrial fission↓	[110]
	SGLT2 Inhibitor	STZ-induced male C57BL/6J mice	Dapagliflozin(10 mg/kg/day) by i.g	ERK1/2↓ cPLA2↓ ROS↓	[125]
	SGLT2 Inhibitor	C57BL/6 male mice,db/db mice	Dapagliflozin (1 mg/kg/day) for 8 weeks	SIRT1↑ eNOS↑ ROS↓	[126]
	SGLT2 Inhibitor	STZ-induced male ApoE^−/−^mice	Canagliflozin (30 mg/kg/day) by i.g	NOX2↓ ICAM-1↓ VCAM-1↓	[127]
	SGLT2 Inhibitor	STZ-induced male ApoE^−/−^mice	Empagliflozin (10 mg/kg/day) by i.g	p-AMPK↑ eNOS↑ mtROS↓	[128]
	GLP-1	STZ-induced male SD rats	rhGLP-1 (1.5pmol/kg/min) by osmotic pumps	PKC-β↓ PKA↑ ROS↓	[120]
	GLP-1	STZ-induced male SD rats	exenatide(5 µg/kg, twice a day) by i.h	NOX4↓ VCAM-1↓	[118]
	Thiazolidinediones	STZ-induced male SD rats	pioglitazone (10 mg/kg) for 4 weeks	SOD↑ CAT↑ GSH↑ ROS↓	[132]
	Thiazolidinediones	STZ-induced male C57BL/6J mice	Rosiglitazone(0.15 g/kg)	HSP22↑ ICAM-1↓	[133]

Abbreviations used are: ↑,upregulation; ↓, downregulation. AMPK, AMP-activated protein kinase; OGG1, 8-Oxoguanine glycosylase; ROS, reactive oxygen species; mtROS, mitochondrial ROS; Tom, translocase of the outer membrane; NO, nitric oxide; GSH, glutathione. DPP-4, dipeptidyl peptidase‑4; SGLT-2, sodium-glucose cotransporter 2; GLP-1, glucagon-like peptide-1. LOX-1, lectin-like ox-LDL receptor- 1; ICAM-1, intercellular cell adhesion molecule-1; VCAM-1, vascular cell adhesion molecule 1; NOX4, nicotinamide adenine dinucleotide phosphate oxidase 4; STZ, Streptozotocin; Drp1, dynamin-related protein 1; TNF-α, tumor necrosis factor α; IL-1β, interleukin-1β; PKA, protein kinase A; eNOS, endothelial nitric oxide synthase; SIRT1, Sirtuin1; PAI-1, plasminogen activator inhibitor-1

### Antioxidants

**Fig. 4:**
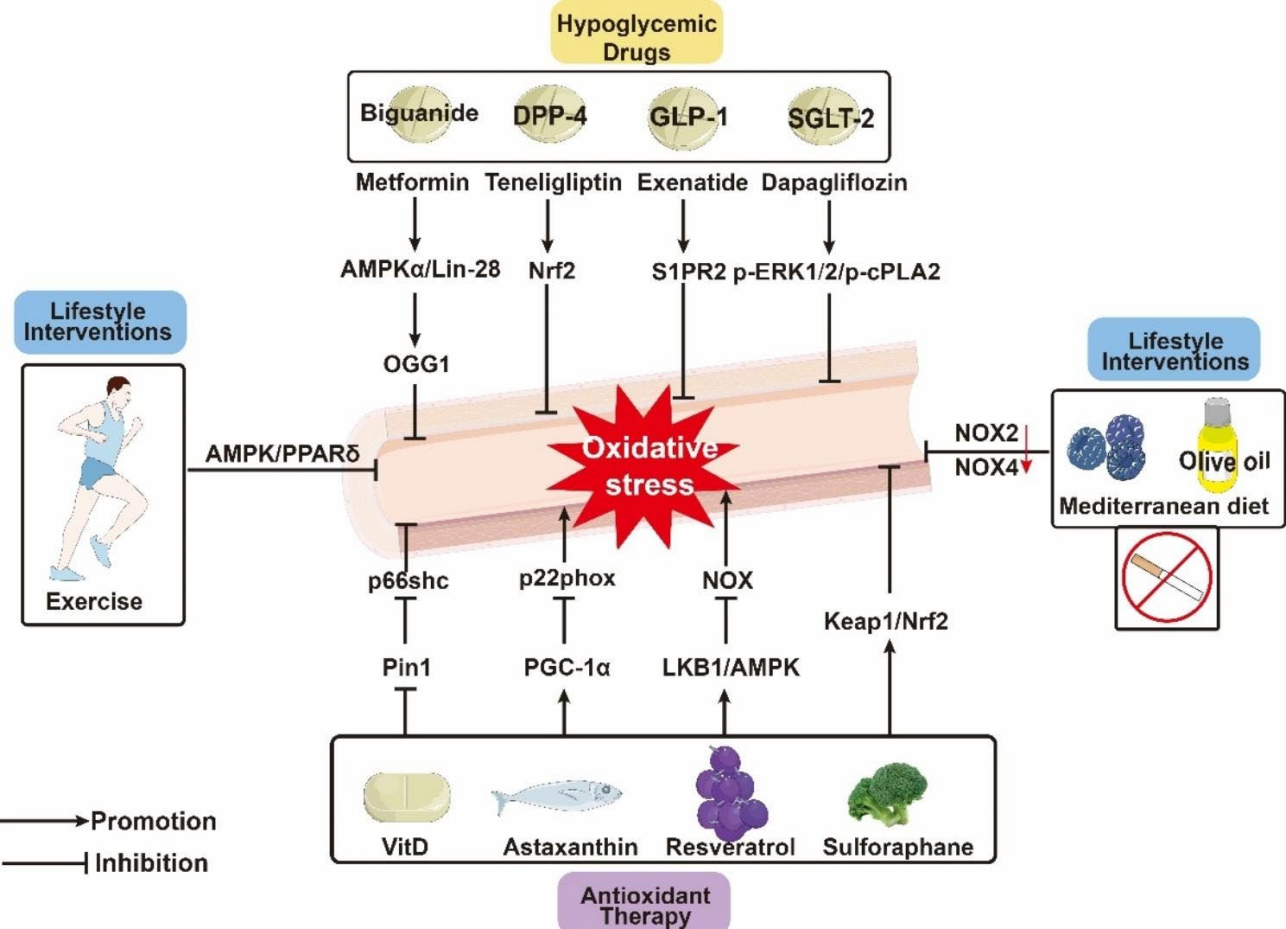
Treatment of diabetic endothelial dysfunction targeting oxidative stress. Hypoglycemic drugs, antioxidant drugs, dietary interventions and lifestyle reduce oxidative stress to improve endothelial function through different pathways. Abbreviations:ROS, reactive oxygen species; Nox, Nicotinamide adenine dinucleotide phosphate oxidase; AMPK, AMP-activated protein kinase; OGG1, 8-Oxoguanine glycosylase; DPP-4, dipeptidyl peptidase‑4; SGLT2, Sodium-glucose Transporter2; ERK1, Extracellular Signal Regulated Kinase 1; GLP-1, glucagon-like peptide-1; S1PR2, sphingosine-1-phosphate receptor 2; Keap, kelch-like ECH-associated protein; Nrf2, Nuclear factor erythroid 2-related factor 2; LKB1, liver kinase B1; Pin1, peptidyl-prolyl cis/trans isomerase 1; PPARδ, peroxisome proliferator-activated receptor δ; PGC-1α, PPARγ coactivator (PGC)-1α.

Some studies have shown that exogenous supplementation with vitamin D3 (VD3) enhances endothelial function by decreasing oxidative stress and increasing antioxidant enzyme activity in the aortas of diabetic rats while significantly reducing fasting blood glucose levels [135] (Fig. 4).

Some studies have revealed that the antioxidative mechanism of VD may involve reducing the expression and activity of Pin1 protein by activating VDR, preventing the Ser-36 phosphorylation and mitochondrial translocation of p66Shc induced by PKC-β, inhibiting mitochondrial oxidative stress and inflammation, and improving endothelial function [136]. VD3 reduces glucose-induced ROS production in RMECs and downregulates TRX-interacting protein expression and NLRP3 activation. In a diabetes-induced retinal vascular injury model, VD3 reduced retinal microvascular apoptosis and vascular permeability and exerted a protective effect against retinal vascular injury and apoptosis [137]. Additionally, the combination of CrPic and VD3 significantly decreased the levels of homocysteine and MDA in T2DM patient blood and enhanced endothelial function [138]. The administration of 1α,25-dihydroxyvitamin D3 (a hormonal form of VD) to vascular endothelial cells cultured in vitro promoted the expression of angiogenic markers and promoted angiogenesis by inhibiting oxidative stress and excessive autophagy through AGE-RAGE interactions and the PI3K/Akt pathway [139].

Resveratrol is a naturally occurring polyphenolic compound that acts as a direct antioxidant, scavenges ROS/RNS and their secondary organic radicals, regulates cellular antioxidant pathways and balances the cellular redox state [140]. In diabetic mice, resveratrol could reduce the expression of Nox2 and p67 by activating the LKB1-AMPK signalling pathway, which subsequently inhibited oxidative stress and attenuated endothelial dysfunction [141]. Likewise, resveratrol increased serum adiponectin levels, reduced proteinuria, and alleviated glomerular mesangial expansion and glomerular cell apoptosis in db/db mice. In addition, resveratrol could reduce high glucose-induced apoptosis in human glomerular endothelial cells by inhibiting oxidative stress by upregulating the expression of AdipoR1 and AdipoR2 and promoting the AMPK-SIRT1-PGC-1α axis and PPARα signalling [142]. In addition to studies on cell and mouse models, resveratrol supplementation in T2DM patients improved glycaemic control by reducing insulin resistance, which significantly alleviated chronic inflammation and oxidative stress in diabetic patients [143].

Carotenoids are a large class of lipid-soluble molecules that have antioxidant and anti-inflammatory effects and contribute to the restoration of endothelial homeostasis by promoting NO bioavailability in vascular endothelial cells chronically exposed to high glucose [144]. Astaxanthin (ASX), a luteolin carotenoid, can reverse the suppression of PGC-1α and elevation of p22phox caused by glucose fluctuations, resulting in a decrease in ROS production in HUVECs; this factor can also reduce endothelial cell apoptosis and downregulate the expression of proinflammatory mediator genes, protecting endothelial cells from the effects of glucose fluctuations [145].

Under basal conditions, most cytosolic Nrf2 is bound by Keap1 to form a Keap1-Nrf2 complex, which prevents Nrf2 from entering the nucleus by anchoring to the actin cytoskeleton. When the body is subjected to high levels of oxidative stress, the Keap1/Nrf2 complex dissociates in the cytoplasm, which leads to Nrf2 nuclear localization, where it forms a heterodimer with other transcription factors that recognize and bind to the antioxidant response element region, accelerating gene transcription [146]. Sulforaphane is an isothiocyanate that occurs naturally and is present in cruciferous vegetables such as broccoli. It was reported to prevent human microvascular endothelial cells from high glucose-induced oxidative stress, aortic fibrosis and inflammation in diabetes by inhibiting the binding of Keap1 to Nrf2 [147, 148].

### Lifestyle interventions

The burden of diabetes is closely related to metabolic risk (high body mass index) and behavioural factors (malnutrition, smoking and lack of physical exercise). Therefore, in addition to conventional medications, lifestyle interventions are also important [1]. The blood of smokers typically exhibits impaired antioxidant capacity and an increase in oxidized lipids. Nicotine, a significant component of e-cigarettes and cigarettes, triggers vascular APN resistance and diabetic endothelial dysfunction through socs3-mediated degradation of AdipoR1 ubiquitination [149]. Cigarette smoke, in turn, exacerbates diabetic vascular fibrosis and induces eNOS uncoupling, enhancing aortic Nox2 and Nox4 expression, which leads to the dysregulation of vascular tone [150]. E-cigarette-induced overproduction of ROS reduces NO bioavailability and increases apoptosis in vascular endothelial cells, further exacerbating endothelial dysfunction and impaired wound healing in diabetic patients [151].

In contrast to long-term chronic hyperglycaemia, acute blood glucose fluctuations can induce oxidative stress, increasing monocyte adhesion to endothelial cells, increasing endothelial cell apoptosis and exacerbating vascular injury [152]. In contrast, regular or intermittent exercise improves oxidative stress biomarkers and delays or prevents the development of prediabetes into diabetes [153, 154]. In diabetic mice, four weeks of exercise was shown to reduce ERS and oxidative stress by inducing AMPK-PPARδ activation, increasing NO bioavailability in endothelial cells and vascular tissue, improving vascular endothelial function, and providing a potentially effective target for the treatment of diabetic vasculopathy [155].

The Mediterranean diet, which is a nutritional pattern centred on fruits, vegetables, legumes, fish, grains and olive oil, has beneficial effects on endothelial cells. Eating more soy nuts improves glycaemic status, increases total serum antioxidant and lipid levels and reduces E-selectin, thus improving endothelial function in T2DM patients [156]. Hydroxytyrosol (HT), which is the primary polyphenol in olive oil, is synthesized with NO to form hydroxytyrosol nitric oxide (HT-NO), which reduces high glucose-induced oxidative stress in HUVECs by upregulating SIRT1 expression and increases NO levels to improve endothelium-dependent vasodilation. In addition, HT-NO has hypoglycaemic and antioxidant effects on DM mice [157]. Supplementation with dietary nitrates and blueberries reduces diabetic endothelial dysfunction and improves vascular dysfunction by reducing Nox-derived oxidative stress [158, 159].

A high-fat, low-carbohydrate diet called the ketogenic diet encourages the generation of ketone bodies. Ketone bodies induce the expression of Nrf2 and HO-1 in the antioxidant defence system in endothelial cells, significantly reducing DNA damage [160]. The main component of ketone bodies, β-hydroxybutyric acid, promotes Cu/Zn-SOD production in diabetic rats and high glucose-treated CMECs and decreases peroxynitrite levels, thereby reducing oxidative stress, inhibiting myocardial microvascular collagen 4 accumulation and antagonizing cardiac microvascular fibrosis in diabetic patients [161].

## Conclusion

This review summarizes the four sources of ROS production in diabetic vascular endothelial cells and the underlying molecular mechanisms by which the diabetic pathogenic factors such as hyperglycemia, hyperlipidaemia, adipokines and insulin resistent induce oxidative stress in endothelial cells of diabetes. In addition, this review provides in vitro and in vivo evidences for protective effects of the oxidative stress-targeted interventions including hypoglycemic drugs, antioxdants and lifestyle interventions on diabetes-induced endothelial dysfunction. Despite of so many basic studies to verify the effectiveness of anti-oxidative stress treatment in hyperglycemia-treated endothelial cells and diabetic mouse models, few of the clinical results support the widespread use of antioxidants in the treatment of diabetic vascular complications. Therefore, novel delivery methods for antioxidants such as nanomaterials [162] or multiple targets-targeted antioxidant strategies should be used in the future clinical studies for diabetic patients with microvascular and macrovascular complications.

## Data Availability

Not applicable.

## Abbreviations

AP-1: Activator Protein-1
APN: Adiponectin
AGEs: Advanced glycosylation end products
Ang-1: Angiopoietin-1
ACE2: Angiotensin-converting enzyme 2
AR: Aldose reductase
ASX: Astaxanthin
CMECs: Cardiac microvascular endothelial cells
CRP: C reactive protein
DM: Diabetes mellitus
DAG: Diacylglycerol
DPP-4: Dipeptidyl peptidase 4
EDHF: Endothelium-Derived Hyperpolarizing Factor
ET-1: Endothelin-1
eNOS: Endothelial nitric oxide synthase
EMPs: Endothelial cell-selective adhesion molecule
ERS: Endoplasmic-reticulum stress
FFA: Free fatty acids
ESAM: Endothelial microparticles
GFAT: Fructose-6-phosphate amidotransferase
GLP-1: Glucagon-like peptide 1
glyLDL: Glycated low-density lipoproteins
HO-1: Heme oxygenase-1
HBP: Hexosamine pathway
HCAECs: Human coronary artery endothelial cells
HECs: Human endothelial cells
HUVECs: Human Umbilical Vein Endothelial Cells
HRVECs: Human Retinal Vascular Endothelial Cells
HRMECs: Human retinal microvascular endothelial cells
HT: Hydroxytyrosol
HT-NO: Hydroxytyrosol nitric oxide
HOCl: Hypochlorous acid
8-OHdG: Hydroxy-2′-deoxyguanosine
ICAM-1: Intercellular cell adhesion molecule-1
IL-1β: Interleukin-1β
Keap1: Kelch-like ECH-associated protein 1
LOX-1: Lectin-like oxLDL receptor-1
MAPK: Mitogen-activated protein kinase
MDA: Malondialdehyde
Mitochondrial ETC: Mitochondrial electron transport chain
mtROS: Mitochondrial reactive oxygen species
NADPH: Nicotinamide dinucleotide phosphate
Nox: NADPH oxidase
NO: Nitric oxide
NOS: Nitric oxide synthase
NF-κB: Nuclear factor kappa B
NRF2: Nuclear factor erythroid 2-related factor 2
NLRP3: NOD-like receptor family, pyrin domain-containing 3
O-GlcNAc: O-linked N-acetylglucosamine
oxLDL: Oxidized low-density lipoproteins
OGG1: Oxoguanine DNA glycosylase
PAI-1: Plasminogen activator inhibitor-1
PXDN: Peroxidasin
PVAT: Perivascular adipose tissue
PPARδ: Peroxisome proliferator-activated receptor δ
PKC: Protein kinase C
ROS: Reactive oxygen species
RAGE: Receptor for AGEs
rhGLP-1: Recombinant human glucagon-like peptide-1
RECs: Retinal endothelial cells
SELENOS: Selenoprotein S
SP1: Specificity protein 1
SOD: superoxide dismutase
SIRT1: Sirtuin 1
SGLT2: Sodium-glucose Transporter2
S1PR2: Sphingosine-1-phosphate receptor 2
SARS-CoV-2: Severe acute respiratory syndrome coronavirus 2
TNF-α: Tumor necrosis factor α
UDP-GlcNAc: UDP-N acetylglucosamine
VCAM-1: Vascular cell adhesion molecule-1
VEGF: Vascular endothelial growth factor
VWF: Von Willebrand factor
VD3: Vitamin D3
XO: Xanthine Oxidase
